# Effect of Temperature-Sensitive Poloxamer Solution/Gel Material on Pericardial Adhesion Prevention: Supine Rabbit Model Study Mimicking Cardiac Surgery

**DOI:** 10.1371/journal.pone.0143359

**Published:** 2015-11-18

**Authors:** Hyun Kang, Yoon Sang Chung, Sang Wook Kim, Geun Joo Choi, Beom Gyu Kim, Suk Won Park, Ju Won Seok, Joonhwa Hong

**Affiliations:** 1 Department of Anesthesiology and Pain Medicine, Chung-Ang University College of Medicine, Seoul, Korea; 2 Heart Research Institute, Chung-Ang University College of Medicine, Seoul, Korea; 3 Department of Internal Medicine, Chung-Ang University College of Medicine, Seoul, Korea; 4 Department of Thoracic and Cardiovascular Surgery, Chung-Ang University College of Medicine, Seoul, Korea; 5 Department of Surgery, Chung-Ang University College of Medicine, Seoul, Korea; 6 Department of Radiation Oncology, Chung-Ang University College of Medicine, Seoul, Korea; 7 Department of Nuclear Medicine, Chung-Ang University College of Medicine, Seoul, Korea; Centro Cardiologico Monzino, ITALY

## Abstract

**Objective:**

We investigated the mobility of a temperature-sensitive poloxamer/Alginate/CaCl_2_ mixture (PACM) in relation to gravity and cardiac motion and the efficacy of PACM on the prevention of pericardial adhesion in a supine rabbit model.

**Methods:**

A total of 50 rabbits were randomly divided into two groups according to materials applied after epicardial abrasion: PACM and dye mixture (group PD; n = 25) and saline as the control group (group CO; n = 25). In group PD, rabbits were maintained in a supine position with appropriate sedation, and location of mixture of PACM and dye was assessed by CT scan at the immediate postoperative period and 12 hours after surgery. The grade of adhesions was evaluated macroscopically and microscopically two weeks after surgery.

**Results:**

In group PD, enhancement was localized in the anterior pericardial space, where PACM and dye mixture was applied, on immediate post-surgical CT scans. However, the volume of the enhancement was significantly decreased at the anterior pericardial space 12 hours later (*P* < .001). Two weeks after surgery, group PD had significantly lower macroscopic adhesion score (*P* = .002) and fibrosis score (*P* = .018) than did group CO. Inflammation score and expression of anti-macrophage antibody in group PD were lower than those in group CO, although the differences were not significant.

**Conclusions:**

In a supine rabbit model study, the anti-adhesion effect was maintained at the area of PACM application, although PACM shifted with gravity and heart motion. For more potent pericardial adhesion prevention, further research and development on the maintenance of anti-adhesion material position are required.

## Introduction

Cardiac surgery is complex and carries a high potential for complications, especially when reoperation is required. Pericardial adhesion from one surgery can greatly complicate subsequent operations. Therefore, adhesion prevention is important, especially in relatively young patients who may require another sternotomy in the future for various indications and in pediatric patients who undergo staged operations [1].

Pericardial adhesion prevention must be handled differently from adhesions in other body parts for several reasons. Unlike other organs, the heart is an actively moving anatomic structure. Moreover, pericardial adhesion must be prevented in all three-dimensional planes in both original and reconstructed anatomic structures, such as coronary bypass grafts, patches, conduits, and great vessels[1].

In the last several years, anti-adhesion materials in many forms such as membranes, solutions, gels and even red wine have been investigated using animal models[1–22]. However, it is not known which form is the most effective for pericardial adhesion prevention after cardiac surgery[13]. In addition to effectiveness, to be applied in clinical fields, the technical ease of such procedures is also an important factor to be considered. Some membrane-type anti-adhesion materials require additional fixing stitches to prevent migration from the original position. Other membrane-type anti-adhesion materials become sticky after application, making repositioning difficult[5, 14, 16]. In contrast to membrane-type materials, solution or gel materials can be easily applied to actively moving three-dimensional structures such as the heart [1, 5, 16]. However, there are concerns on whether solution or gel anti-adhesion materials remain where they are first applied or if they move to other locations because of heart motion and gravity.

In most animal studies performed to study pericardial adhesion prevention with solution or gel materials, animals were kept in their natural prone position after the surgical procedure[1–6, 12, 14, 15, 19]. However, this position differs from that used in clinical settings. In these prone position animal studies, it was assumed that most of the solution or gel type anti-adhesion materials collected in the retrosternal area where macroscopic adhesion grading was performed and tissue was sampled for microscopic adhesion grading. However, in clinical settings, patients are in the supine position for several hours after cardiac surgery. In the supine position, gravity works opposite to the retrosternal area in the pericardial space. Because of the opposing directions of gravity and heart pumping motion, recent animal studies performed with prone positioning cannot be applied to patients in clinical settings. Additionally, results of the animal studies might have overestimated retrosternal anti-adhesion effects.

Temperature-sensitive poloxamer/alginate/CaCl_2_ mixture (PACM), a solution-gel type anti-adhesion material, has been shown to be effective for pericardial adhesion prevention in prone position animal study[1]. In the present study, we used a supine rabbit model to mimic the clinical setting of cardiac surgery and CT scans to study whether PACM was retained at the site of application or if it shifted to another location due to gravity and cardiac motion in a supine position. We also studied the efficacy of PACM on the prevention of pericardial adhesion.

## Material and Method

### Stability of the Poloxamer/Alginate/CaCl_2_ Mixture and Radio-opaque Dye Mixture

**Fig 1:**
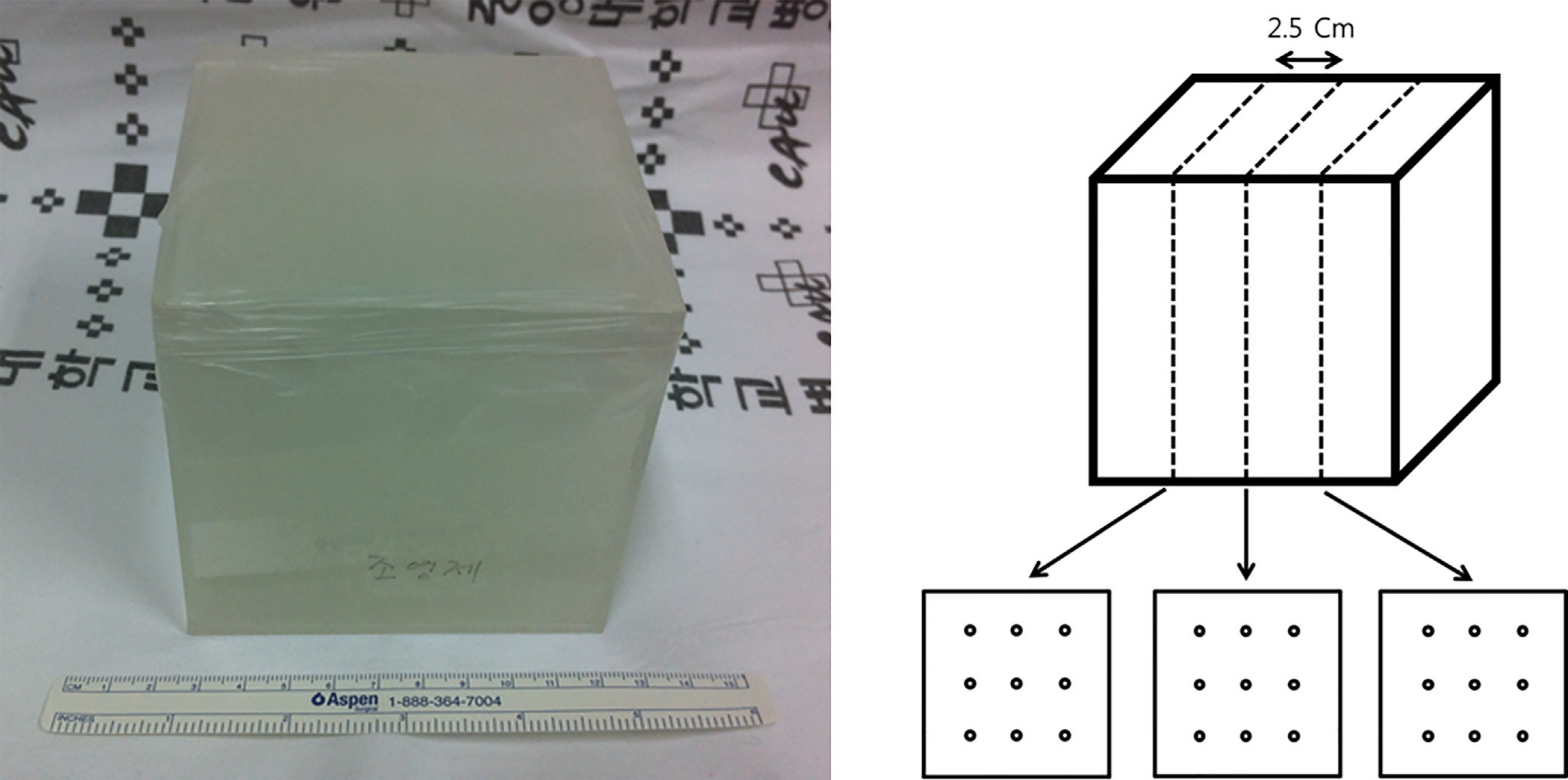
Picture of a container with the PACM and dye mixture (left panel). The containers with the mixture were scanned at a distance of 2.5ⅹ10^−2^ m. A total of 27 circles (nine circles on each of three sections) with 1.0ⅹ10^−2^ m diameter were drawn on each scanned image (right panel). Hounsfield units were recorded in each circle.

To verify the homogeneity and stability of PACM and radio-opaque iodixanol dye (Visipaque, GE Health Care, Korea), we made six mixtures of 1 L of PACM and 2.0ⅹ10^−3^ L of radio-opaque dye and placed them in six 1.0ⅹ10^−3^ m^3^ containers. We performed CT scans (Lightspeed RT, GE Healthcare, USA) of the containers and recorded Hounsfield units at 27 equidistant spots (Fig 1) in each container. After scanning, three containers were kept at room temperature (22°C), and the other three containers were maintained at body temperature (37°C) for 24 hours. After this time period, new CT scans of all six containers were obtained, and Hounsfield units were compared with those previously measured.

### Study Animals

This study was performed in the Animal Research Laboratory of Chung-Ang University Hospital and was approved by the Chung-Ang University Institutional Review Board of Animal Research(IRB No. C 14–0047). All procedures were performed in accordance with the National Institute of Health Guide for the Care and Use of Laboratory Animals. A total of 50 New Zealand white rabbits (2.5–3.0 kg each; Dayoon, Seoul, Korea) were used. All rabbits were housed two per cage in a temperature-controlled room (22°C) under a 12-h light/12-h dark cycle and were fed a standard laboratory diet and tap water. The physical condition of the animals were monitored twice per day for possible surgical complications.

### Surgical Technique

All procedures were performed using the technique previously described [1], in sterile conditions by the same surgical team, which were previously described Anesthesia was provided by intramuscular injection of 25.0 mg/kg ketamine hydrochloride (Ketamine Hcl; Huons, Seoul, Korea). After appropriate sedation was achieved, an intravenous line was installed. Intravenous cefazolin (Chong Kun Dang Pharmaceutical Corp., Seoul, Korea) at 30 mg/kg was given as a prophylactic antibiotic. With endotracheal intubation, general anesthesia was maintained using 1–3 vol% isoflurane with mechanical ventilation. The anterior chest was shaved and cleaned with povidone iodine for a median sternotomy. The skin incision was made and deepened to the sternum. The sternum was divided with straight scissors taking care not to open the pleura. The pericardium was opened longitudinally 3–4 cm to expose the anterior wall of the heart. The exposed epicardial surfaces were desiccated and abraded for 3 minutes with gauze to induce approximately 2.0ⅹ2.0ⅹ10^−4^ m^2^ adhesions.

Rabbits were allocated into one of two groups based on a random table generated using PASS 11 (NCSS, Kaysville, Utah, USA). The randomization sequence was generated by a statistician who was not involved in the study design. Details of the series were unknown to the surgeons who performed the procedures, and the group assignments were kept in sealed envelopes labeled with only the case number. To keep the surgeon “blind” to the assigned group, the appropriate numbered envelope was opened just before applying the PACM and dye or normal saline; the card inside determined whether the patient would be in group PD or group CO.

In the PACM and dye group (group PD, n = 25), 1.0 mg/kg of the PACM and radio-opaque dye mixture was applied on the anterior surface of the heart over the desiccated area. In the control group (group CO, n = 25), 1.0 mg/kg of saline was applied. Once the treatment was applied, two Prolene 5–0 stitches were placed to close the pericardium and mark the site of the abrasion. If the pleura was opened, a 10-Fr trocar chest tube was placed through the intercostal space, and a purse string suture was placed around the chest tube. The chest tube was removed while connected to gentle suction, and the lungs were inflated by positive ventilation using an Ambu bag. The purse string suture was then tied. The endotracheal tube was removed when self-respiration returned. In group PD, rabbits were maintained in a supine position for 12 hours with appropriate sedation using intermittent ketamine injections. After 12 hours in the supine position, a CT scan was performed in group PD.

Two weeks after surgery, all rabbits in both groups were sacrificed with a lethal dose of ketamine. The heart was exposed through a redo-median sternotomy for macroscopic evaluation of adhesion between the pericardium and the heart. The heart and the pericardium were removed en bloc for microscopic evaluation.

### CT Evaluation

**Fig 2:**
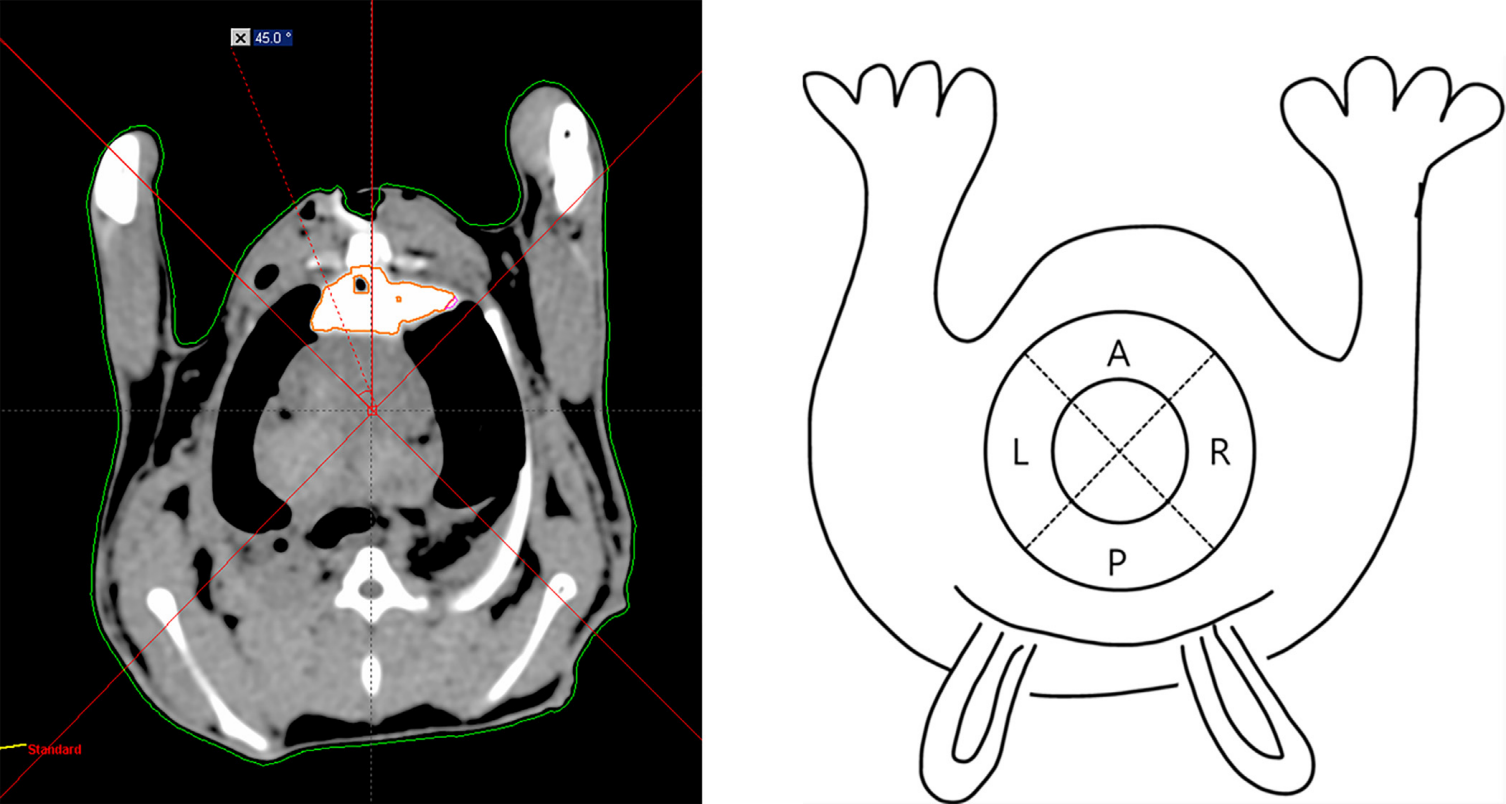
Example of image design for CT evaluation (left panel), and a schematic of a CT scan of four pericardial segments (right panel). A, anterior; P, posterior; L, left; R, right.

In group PD, where rabbits were kept in a supine position, location of the PACM and dye mixture was evaluated by CT scan in the immediate postoperative period and 12 hours after surgery. A baseline between the center of the spine and the sternum was drawn on the CT image using software (Eclipse^TM^, Varian Medical System, Inc., USA). Two additional lines, forming a 45° angle on each side of the baseline, were drawn to divide the pericardial space into anterior, posterior, right, and left segments (Fig 2). We considered Hounsfield units between 160 and 1667 in the pericardial space as enhancement due to the PACM and dye mixture. We measured the total volume of enhancement in the pericardial space and the volume of enhancement in each pericardial segment.

### Macroscopic Evaluation

Two surgeons who were blinded to the study purpose scored the macroscopic adhesions separately, and a consensus score was obtained for each rabbit. The adhesion score system described in Table 1 was used to determine the severity of adhesion.

**Table 1:** Adhesion Score System for Macroscopic Evaluation.

Score	Description
0	No adhesion
1	Mild adhesion, easy to dissect manually
2	Moderate adhesion, cohesive and can be dissected manually
3	Severe adhesion, cohesive and sharp dissection required
4	Very severe adhesion, cohesive adhesion requiring aggressive dissection that damages adherent tissue

### Microscopic Evaluation

**Fig 3:**
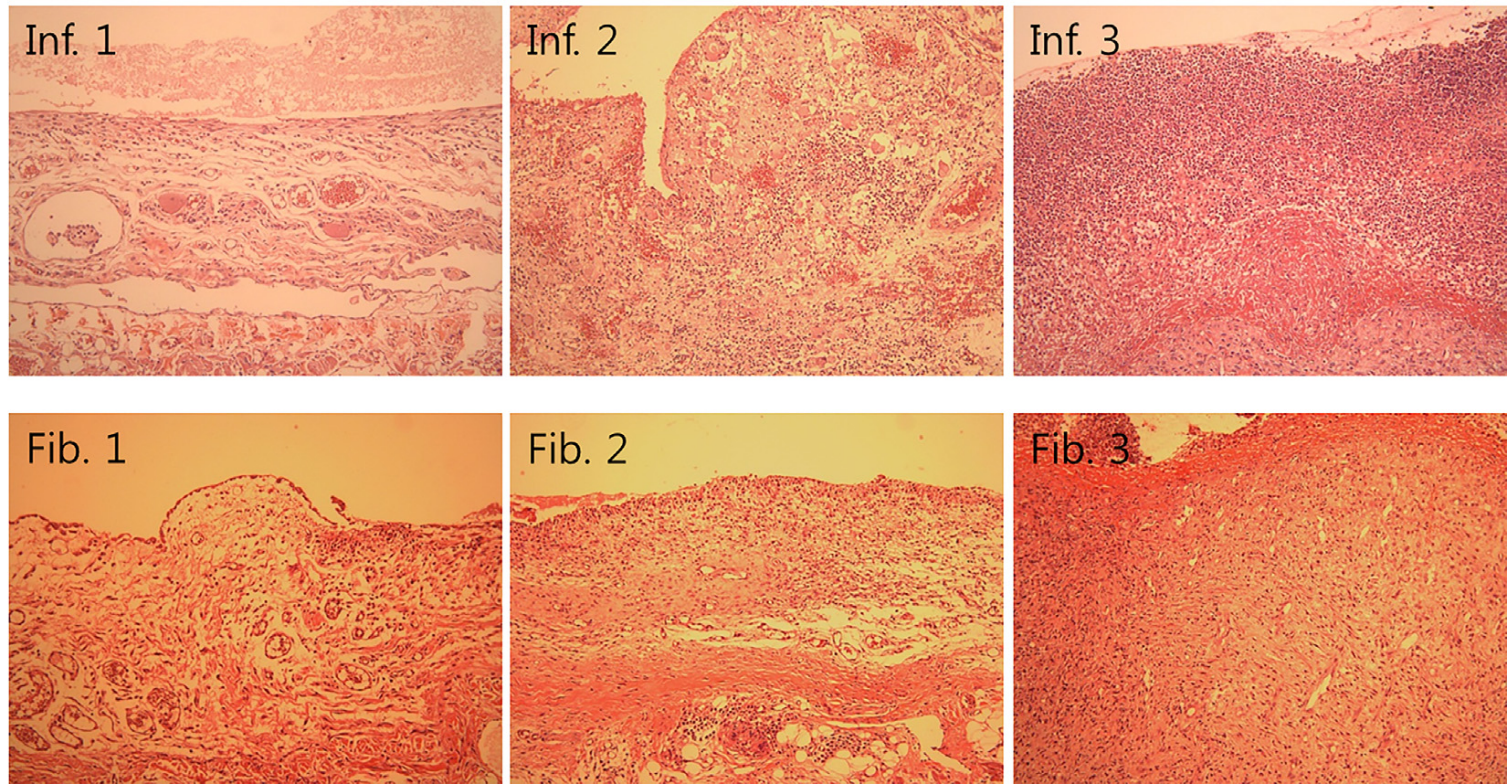
Histologic examination of inflammation and fibrosis (H&E stain, X 200). (Inf. 1) Inflammation grade 1: mild lymphocytic infiltration with several foreign body giant cells; (Inf. 2) Inflammation grade 2: moderate infiltration of neutrophils, eosinophils, and foreign body giant cells; (Inf. 3) Inflammation grade 3: marked neutrophil infiltration (abscess) along the pericardial surface. (Fib. 1) Fibrosis grade 1: loose fibrosis beneath the pericardial surface; (Fib. 2) Fibrosis grade 2: moderate fibrosis beneath the pericardial surface; (Fib. 3) Fibrosis grade 3: marked fibrosis beneath heavy leukocytic infiltration.

Microscopic evaluation was performed as previously described [1]. After macroscopic scoring, segments of the pericardium and heart, especially from the site of abrasion marked with a Prolene stitch, were fixed in 10% neutral buffered formalin for 24 h and stained with hematoxylin and eosin. All immunohistochemical analyses were performed using an automatic immunostaining device (Autostainer 480; Labvision, Fremont, CA). Next, 5-mm tissue sections were exposed to 10 mM citrate buffer (pH 6.0) and heated for 20 min in a water bath for antigen retrieval. In order to evaluate anti-macrophage antibody (AMA) expression, tissue sections were incubated for 60 min with mouse monoclonal antibody against rabbit macrophages (clone 3H2617, dilution 1:100; Santa Cruz Biotechnology Inc., Santa Cruz, CA). Antibody binding was detected using the HRP polymer conjugate (TL-125-HL kit; Labvision) and visualized using the 3-amino-9-ethylcarbazole (AEC) chromogen. Sections were counterstained with Gill’s hematoxylin solution and mounted in aqueous mounting media. The degrees of fibrosis and inflammation were evaluated using a semiquantitative scoring system (Fig 3). Fibrosis and inflammation scores are given in Table 2. The number of immunopositive cells was counted three times at 3200x magnification, and the results were averaged.

**Table 2:** Microscopic Adhesion Score System.

Score	Fibrosis grade	Inflammation grade
0	None	None
1	Minimal, loose	Giant cells, lymphocytes, plasma cells
2	Moderate	Giant cells, eosinophils, neutrophils
3	Florid dense	Many inflammatory cells, microabscess

### Statistical Analysis

The primary outcome measure of this study was the post-operative macroscopic adhesion score. To calculate the sample size required for this study, macroscopic adhesion scores from a previous study were taken into account[1]. The mean and standard deviation of the macroscopic adhesion score in the control group of that study was 2.79 ± 0.79. For our power calculation, we assumed an equal standard deviation in group PD. We wanted the capability to show a 25% reduction in the macroscopic adhesion score. With an α = .05, two-tailed t-test and a power of 80%, we needed 21 rabbits per group. Considering a compliance rate of 85%, we allocated 25 rabbits to each group.

The normal distribution of the collected data was first evaluated using the Shapiro-Wilk test. Normally distributed data were analyzed using parametric tests, and abnormally distributed data were analyzed using nonparametric tests. As AMA expression was normally distributed, between-group comparison was evaluated by student-t test. As macroscopic adhesion, fibrosis, inflammation and volumes measured by CT were abnormally distributed, between-group comparisons were evaluated using Mann-Whitney U test and within-group comparisons were evaluated using Wilcoxon signed rank test.

Associations among the macroscopic adhesion scores, fibrosis/inflammation scores, and AMA expression were evaluated using the Spearman rank correlation coefficient (ρ). Inter-observer agreement for macroscopic adhesion scores were evaluated using Kappa analysis. The stability of the PACM and radio-opaque dye mixture was evaluated using the Friedman test.

## Results

### Stability of the Poloxamer/Alginate/CaCl_2_ Mixture and Radio-opaque Dye Mixture

CT scans of the mixtures in all six containers showed homogenous opacity. All 27 spots in each container showed the same Hounsfield units (*P* = .327) and did not change after 24 hours (*P* = .867).

Having verified the stable homogeneity of the mixture at both temperatures, we assumed that the mixture distribution in the pericardial space of the rabbits could be evaluated by CT scan.

### Study Animals

There were five rabbit death during the study. Two rabbits in each group died within two weeks due to infection, and one rabbit in group PD died of possible pneumothorax. A total of 45 rabbits (22 in group PD, 23 in group CO) survived for two weeks.

### CT Evaluation

**Fig 4:**
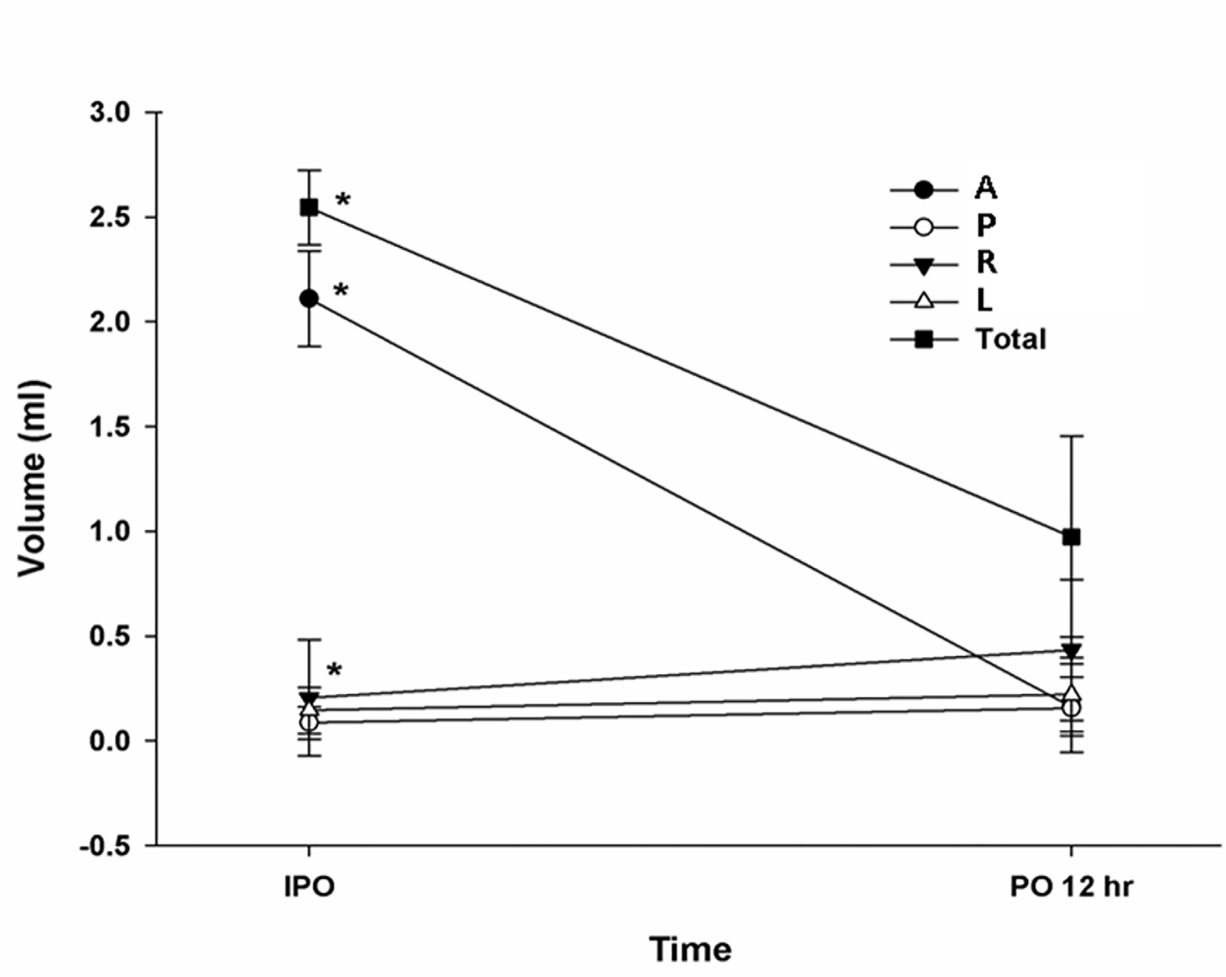
Changes in measured volume at each segment. A, anterior; P, posterior; L, left; R, right; IPO, immediately postoperative; PO 12 hr, postoperative 12 hours.

In group PD, 3.38 ± 0.39 mL of the PACM and dye mixture was applied. There was good correlation between total volume of applied mixture and the total volume measured by CT scan immediately after the surgery (2.73 ± 0.61ⅹ10^-3^L) (ρ = .852, *P* < .001) (Fig 4).

Most of the enhancement was localized at the applied position (the anterior pericardial segment) on the CT scan immediately after the surgery (2.10 ± 0.68ⅹ10^-3^L). However, 12-hours postoperatively, the volume of the enhancement was significantly decreased in the anterior pericardial segment (from 2.10 ± 0.68 to 0.46 ± 0.50 ⅹ10^-3^L, *P* < .001) and increased at the right segment (from 0.29 ± 0.06 to 0.50 ± 0.21 ⅹ10^-3^L, *P* < .001). However, we found no significant changes in the left or posterior segments (from 0.11 ± 0.05 to 0.12 ± 0.07 ⅹ10^-3^L, *P* = .564; from 0.23 ± 0.17 to 0.30 ± 0.02 ⅹ10^-3^L, *P* = .060, respectively) (Fig 4).

The total volume of the enhancement measured on the CT scan decreased significantly 12 hours later (from 2.73 ± 0.61 to 1.37 ± 0.66 ⅹ10^-3^L, *P* < .001) (Fig 4).

### Macroscopic Evaluation

**Fig 5:**
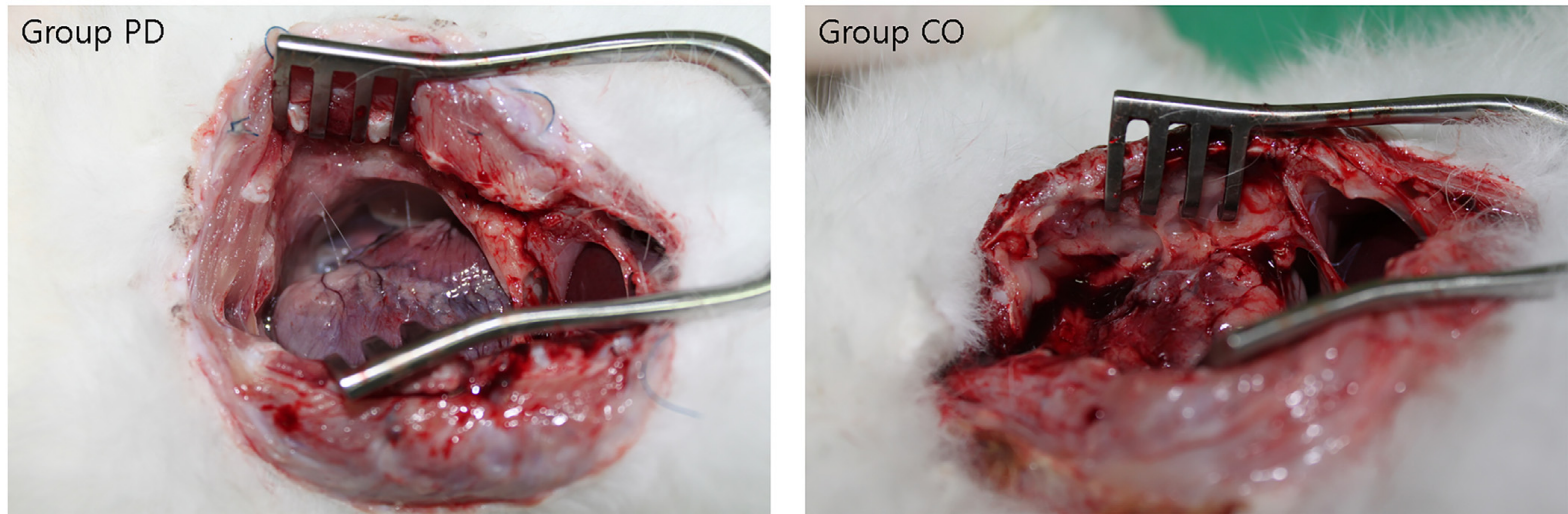
Macroscopic evaluation. A rabbit with macroscopic adhesion score 0 in group PD (left panel) and score 4 in group CO (right panel).

The adhesion score in group CO was 2.59 ± 1.18, while that in group PD was 1.43 ± 0.84. Group PD had a significantly lower macroscopic adhesion score (Fig 5) compared to group CO (*P* = .002), as shown in Table 3. Inter-observer agreement for macroscopic adhesion score proved to be substantial (K = 0.698, P<0.001).

**Table 3:** Macroscopic and Microscopic Adhesion Score.

	Group CO (n = 22)	Group PD (n = 23)	*P* value
Macroscopic adhesion score	2.59 (1.18)	1.43 (0.84)	.002* †
Inflammation score	1.77 (0.75)	1.30 (0.76)	.093*
Fibrosis score	1.55 (0.60)	0.96 (0.83)	.018* †
Anti-macrophage antibody level	14.14 (9.26)	10.64 (6.23)	.147

Data are given as mean with SD in parentheses.

*Mann-Whitney U test because of abnormal distribution.

† *P* < .05 vs. group CO

### Microscopic Evaluation

Two weeks after surgery, fibrosis, inflammation, and the expression of AMA were evaluated microscopically, as shown in Table 3. The fibrosis score was significantly lower in group PD than in group CO (*P* = .018). The inflammation score and expression of AMA in group PD were also lower than those in group CO, although differences were not significant.

## Discussion

PACM is a barrier material that has been shown to have anti-adhesion effects for many different organs [1, 17, 21]. However, prevention of pericardial adhesion is different from that of other organs, primarily because of the active motion of the heart and gravity working against the retrosternal area when in a supine position. Although several anti-adhesion materials have been shown to be effective for pericardial adhesion prevention in animal studies, there is no human data to date. Human study is difficult to perform because of the technical difficulty of long-term follow-up, especially among adult cardiac surgery patients. Therefore, studies on pericardial adhesion prevention have relied on well-designed animal studies and detailed interpretation of results. However, there have been no previous reports performed under conditions mimicking the human clinical environment: sternotomy and a supine position during the postoperative period.

In the past, various studies have been performed to investigate anti-adhesion effects of various pharmacologic agents such as anticoagulants, antibiotics, anti-inflammatory agents, fibrinolytic agents, and antioxidant agents[2–4, 15, 19]. In addition to pharmacologic agents, several natural barriers and synthetic physical barriers have been studied to achieve anti-adhesive effects in different places in the body. The pericardium, peritoneum, omentum, and amnion have been studied and found to be useful as natural barriers [7, 23]. Synthetic physical barriers such as silicone, polytetrafluoroethylene, cellulose, polyvinyl alcohol, polyester derivatives, and collagen membrane have also been studies in various anatomical spaces including the pericardial space [1, 2, 5–8, 10–13, 16–18, 20, 21]. Combinations of pharmacologic and barrier materials have been also attempted in previous studies[14, 22].

The basic function of barrier material is separation of the injured tissue surface from the adjacent tissue[24]. There are many forms of barrier-type anti-adhesion materials, such as membranes, solutions, and gels.

Membrane barriers composed of many different materials are commercially available. However, because of technical difficulties, the possibility of migration, difficulty with repositioning, and challenges of covering three-dimensional structures such as the heart, cardiac surgeons understand the disadvantages associated with these materials and are reluctant to use them [1, 14, 16]. Moreover, additional stitches used to fix the membrane barrier to anatomical structures can cause further adhesions at stitch sites.

There are some commercially-available gel-type barriers. These barriers have an advantage in ease of application to the heart or vessels and. They all have different viscosities, absorption and excretion rates, and anti-adhesive effects [1, 14, 16].

PACM is a poloxamer-based agent cross-linked with alginate and CaCl_2_ and is known for its anti-adhesion effects [1, 10, 16, 17, 21, 22]. The difference in viscosity at room temperature (2960 cP at 22°C) versus body temperature (90,000–95,000 cP at 37°C) is a unique characteristic. At room temperature, PACM is very easily applied to any anatomical surface, and the high viscosity of PACM at body temperature is helpful in maintaining a physical barrier at the original position and preventing adhesion formation [1, 21].

In this study, PACM shifted to other positions, most likely due to the heart’s motion and gravity. The enhancement volume in the anterior segment decreased significantly 12 hours after the application. Also, enhancement in the right, left, and posterior segments increased 12 hours after the application, although only the right segment volume increase was significant. Those changes in enhancement volume are evidence of PACM shifting.

We also found that PACM moved more to the right side of the heart than to the left. We hypothesize this was because of the lower intrapericardial pressure at the right atrium than at the left ventricle. The main difficulties of redo-sternotomy are opening the sternum and dissecting the great vessels, such as the aorta, superior vena cava, inferior vena cava, and the right atrium, for cannulae insertion. Therefore, the shift of PACM to the right side is advantageous in light of adhesion prevention at venous cannulae insertion sites, although it remains to be determined whether the amount of PACM shifting is directly related to its anti-adhesion effects.

Although PACM shifted to other segments on the CT scan, the macro- and microscopic adhesion scores in the anterior segment are lower in group PD than in group CO, suggesting that a sufficient amount of PACM remained at the anterior segment to remain effective. However, we do not know whether all other commercially available solution or gel materials will remain effective when shifted to other segments. PACM is a material with very high viscosity at body temperature. This study suggests that anti-adhesion solution or gel material with a lower viscosity than PACM would move in accordance with gravity and cardiac motion, leaving an insufficient amount of the anti-adhesion material at the original position.

At this time, it remains unclear which anti-adhesion material in which form is the best for pericardial adhesion prevention. Convenience of use and anti-adhesion effect are both important. In the pericardial space, a suitable material must not disturb the heart motion or drainage of blood through chest tubes, and the material itself must not easily drain through the chest tubes.

Designing and conducting an animal study to adequately mimic the clinical environment is a challenging task. Our study is valuable in that we showed that an anti-adhesion gel shifts in position when study animals remain in a supine position, which more closely resembles a clinical environment. This result suggests that a large portion of solution- or gel-type anti-adhesion material may shift in patients in a clinical setting. Researchers of anti-adhesion materials consider this finding in their analyses. To the best of our knowledge, this is the first study on mobility of anti-adhesion material in the pericardial space. Our animal experiment model is expected to be useful in future investigations.

For the study of pericardial adhesion prevention in animals, many studies, including this one, have performed animal sacrifice for adhesion evaluation two weeks after the initial adhesion-inducing surgery.[1, 3–5, 15, 22] Other investigators have used different postoperative periods (from one day to 300 days) for the performance of re-sternotomy for adhesion evaluation in different animals including rabbits, dogs, rats, sheep and pigs[2, 6–9, 11–13, 18–21]. For cardiac surgeons, two weeks may not be a usual time frame for a reoperation that results in significant difficulties with adhesionolysis in cardiac surgery patients. However, in animal experimental model studies showed that re-operation and adhesion evaluation could be performed successfully two weeks after the initial surgery [5].

Our study had several limitations. First, there was almost no bleeding in this animal study model, which is different from the clinical environment. We assume that bleeding could alter PACM position and adhesion prevention effects. Second, we did not place a mediastinal tube that would drain PACM as chest tubes used in cardiac surgery patients are too big in diameter to be used in rabbit models. But, as shown in this study with CT scan, sufficient layer of PACM may remain effective even after some of PACM is drained through draining catheter. Third, we did not use a cardiopulmonary bypass pump. However, the aforementioned limitations are challenges that most anti-adhesion animal studies experience. As in our study, further study is needed to eliminate such limitations.

In conclusion, in a supine rabbit model mimicking the clinical setting of human cardiac surgery, the anti-adhesion effect was maintained at the application location; however, a mixture using both PACM and dye did not remain in place due to gravity and heart motion. We assume other materials with lower viscosity might show different results. For more potent pericardial adhesion prevention, further research and development into the physical properties of anti-adhesion material that is able to maintain its position are required.
